# Behavioral testing of mice exposed to intermediate frequency magnetic fields indicates mild memory impairment

**DOI:** 10.1371/journal.pone.0188880

**Published:** 2017-12-04

**Authors:** Kajal Kumari, Hennariikka Koivisto, Matti Viluksela, Kaisa M. A. Paldanius, Mikael Marttinen, Mikko Hiltunen, Jonne Naarala, Heikki Tanila, Jukka Juutilainen

**Affiliations:** 1 Department of Environmental and Biological Sciences, University of Eastern Finland, Kuopio, Finland; 2 A. I. Virtanen Institute, University of Eastern Finland, Kuopio, Finland; 3 National Institute for Health and Welfare, Environmental Health Unit, Kuopio, Finland; 4 Institute of Biomedicine, University of Eastern Finland, Kuopio, Finland; Consiglio Nazionale delle Ricerche, ITALY

## Abstract

Human exposure to intermediate frequency magnetic fields (MF) is increasing due to applications like electronic article surveillance systems and induction heating cooking hobs. However, limited data is available on their possible health effects. The present study assessed behavioral and histopathological consequences of exposing mice to 7.5 kHz MF at 12 or 120 μT for 5 weeks. No effects were observed on body weight, spontaneous activity, motor coordination, level of anxiety or aggression. In the Morris swim task, mice in the 120 μT group showed less steep learning curve than the other groups, but did not differ from controls in their search bias in the probe test. The passive avoidance task indicated a clear impairment of memory over 48 h in the 120 μT group. No effects on astroglial activation or neurogenesis were observed in the hippocampus. The mRNA expression of brain-derived neurotrophic factor did not change but expression of the proinflammatory cytokine tumor necrosis factor alpha mRNA was significantly increased in the 120 μT group. These findings suggest that 7.5 kHz MF exposure may lead to mild learning and memory impairment, possibly through an inflammatory reaction in the hippocampus.

## Introduction

Electromagnetic fields are ubiquitous in the environment with new technologies and novel applications being actively developed and commercialized. Numerous studies have addressed health effects of extremely low frequency (ELF) magnetic fields associated with generation, transmission and use of electric power and those of radiofrequency (RF) electromagnetic fields emitted by, e.g., mobile communication systems. However, few data are available on possible health effects of intermediate frequencies (IF) fields between the ELF and RF ranges [1]. The IF range is usually considered to cover frequencies from 300 Hz to 100 kHz (or up to 10 MHz; the upper limit depends on how RF is defined). Human exposure to IF fields is increasing due to applications like electronic article surveillance systems [2] and induction heating cooking hobs [3].

The present study was conducted to assess cognitive and behavioral effects of long-term exposure to IF magnetic fields (MF). Mice were exposed for 5 weeks to 7.5 kHz MFs similar to those emitted by one of the electronic article surveillance technologies commonly used in supermarkets and other stores to protect merchandise against theft. The magnetic flux densities used were 12 and 120 μT. The higher flux density exceeded the International Commission on Non-Ionizing Radiation Protection reference level (100 μT in the frequency range 3 kHz—10 MHz) for occupational exposure [4] and was higher than the maximum exposure levels (up to 60 μT) found around EAS systems used in supermarkets and libraries [2].

Organisms exposed to environmental agents often show functional or behavioral changes prior to structural changes [5]. Therefore, a broad neurological behavioral test battery was used to assess possible effects on mice exposed to IF MF. In addition, we assessed eventual morphological changes by using sensitive cellular biomarkers, adult neurogenesis and astrogliosis. The subgranular zone of hippocampal dentate gyrus (DG) is an active site of neurogenesis throughout life in humans and other mammals [6]. Hippocampal adult neurogenesis is exquisitely sensitive to changes in its microenvironment and external factors such as radiation [7]. The central nervous system responds to diverse neurological injuries with a vigorous activation of microglial cells and astrocytes. The reactive astrocytes may benefit an injured nervous system by participating in diverse biological processes [8]. Therefore, we focused on adult neurogenesis and gliosis as markers of potential IF MF effects on the brain.

## Materials and methods

### Exposure setup

A detailed description of the exposure system, including measurement of field homogeneity and numerical dosimetry (modelling of electric fields induced in animal tissues), is available elsewhere [9]. Briefly, the exposure system consisted of a wooden rack with five rectangular coils. The dimensions of the coils were 40 cm × 120 cm. The top and bottom coils had 6 turns of copper wire (diameter 1.8 mm), while the three middle coils had 4 turns. The vertical distance between the coils was 25 cm. There were 4 shelves for animal cages; each shelf was 4 cm above one of the coils. The 7.5 kHz signal was generated by a Thandar TG501 function generator (Thurlby Thandar Instruments, UK) and amplified with a Behringer Europower EP 4000 amplifier (Music Group Services, USA) capable of producing 1250 W of continuous power per channel. A 1.47 Ω resistor was connected in series with the coil system. As the inductance of the coil system considerably resists flow of current at 7.5 kHz, a capacitor (about 1.5 μF) was added to tune the system into resonance at 7.5 kHz. Three identical exposure system were made (sham, 12 μT and 120 μT). Control animals were sham-exposed in an identical unenergized coil system. Magnetic field measurements were performed with a calibrated single axis field sensing coil (diameter 2.0 cm, length 1.3 cm). The sensing coil was shielded against electric fields and connected to a multimeter (Agilent U1241B, Agilent Technologies, Malaysia) to measure voltage induced in the coil. The measured fields showed an overall homogeneity (SD) of 3.8%, with all measured points within ± 9% of the overall average. The average of measure field strengths was 12.03 μT for the low exposure (12 μT) group. For a mouse near the end of the exposure period (27.4 g; the induced fields increase with body mass), the modelled peak electric field strength in the brain (maximum in any area of the brain, averaged over a cube of 2.4 mm side length) was found to be 48 mV/m in the 120 μT group (4.8 mV/m in the 12 μT group).

The animals were group housed in transparent polypropylene cages (3–10 animals per cage), with maximum dimensions of 39 × 28 × 14 cm at the cage top and minimum dimensions at the bottom of 33 × 23 × 14 cm.

### Animals and experimental design

In total 70 male C57BL/6J mice (Laboratory Animal Center, University of Eastern Finland, Kuopio, Finland) were exposed continuously for 5 weeks to 7.5 kHz magnetic fields at 12 μT (n = 20) or 120 μT (n = 20), or were sham exposed (n = 30). The animals were 2 months old at the onset of exposure. The tests were run in three cohorts. Each of the first two cohorts included 10 sham-exposed mice and 10 mice exposed at 12 μT. Because of technical problems in the exposure setup (malfunctioning magnetic field meter), exposure to the higher MF was performed separately using 10 sham-exposed mice and 20 mice exposed at 120 μT.

Throughout the experiment, animals were group housed in a controlled environment (temperature 21.2 ± 0.7°C, humidity 49.7 ± 10%, illumination 120 ± 70 lux, light period 07:00 AM to 7:00 PM). Food and water were available *ad libitum*. The experiments were conducted according to the European Council (Directive 86/609) and Finnish guidelines, and approved by the Animal Experiment Board in Finland.

The mice were exposed to the IF field continuously for 5 weeks, except for about one hour per week that was needed for changing the cages. The general health of the mice was followed twice weekly during change of the water bottles by observing their posture, fur condition, spontaneous locomotion and reactivity to handling. At the end of the exposure period, the mice were ear marked (by an animal caretaker to leave the researchers blinded as to the individual exposure history during the behavioral testing) and individually housed in metal cages (40 × 24 × 14 cm). Motor functions, anxiety, aggression, and learning and memory were assessed using a battery of well-established behavioral tests during three weeks after termination of the exposure. There was no exposure during the behavioral tests. During the fourth week after termination of exposure (when the behavioral tests had been completed), the animals were weighed and then euthanized with an intraperitoneal injection of pentobarbiturate-chloralhydrate cocktail (105 mg/kg pentobarbiturate and 425 mg/kg chloral hydrate) for collecting tissue samples.

### Behavioral tests

Motor activity and coordination [10], neophobia [11], anxiety [12], aggression [13], and spatial learning and memory [14, 15], were assessed using well-established battery of behavioral tests. All tests started in the morning around 9 A.M. The mice were tested by a female researcher who did not use any strong odorants. The behavioral testing took place in quiet, dimly lit designated testing rooms.

#### a. Spontaneous exploratory activity

This test was performed in an observation cage (26 × 26 × 39 cm) with white opaque walls using an infrared photobeam detection method coupled with an automated activity monitor (TruScan®, Coulbourn Instruments, CO, USA). The system was designed to enable separate monitoring of horizontal (XY-move time) and vertical activity (rearing). The test session took 10 min and was replicated after 48 h to assess the extent of habituation to the test cage. To avoid odor traces the test cage was cleaned with 70% ethanol before each mouse.

#### b. Motor coordination and balance

Animals were tested with the accelerating Rota-Rod® apparatus (Ugo Basile, Comerio, Italy) for motor coordination and balance. The mouse was placed on a round rod (2 cm in diameter), the rotation of which gradually increases from 5 to 30 rpm. The time to fall off the rod (or turn two full rounds around with the rod) was recorded up to a 6-min cut-off time. The mouse was adapted to the test by first giving it 30 s to stay on a stationary rod and additional 30 s with the minimum speed of rotation.

#### c. Marble burying task for object neophobia

The marble burying task was run in the home cage (27 cm x 45 x cm 14.5 cm) by placing an array of 3 x 3 glass marbles (diameter 1 cm) on the bedding and counting the number of visible marbles the next morning. A high number of covered marbles indicates anxiety.

#### d. Novelty suppressed feeding test

The novelty suppressed feeding test was an additional test for anxiety. After 14 hours of fasting, the mouse was placed into a novel cage with a regular food pellet. The time to sniffing and biting was measured. To control for appetite, the mouse was given a fixed portion of standard chow in its home cage and the amount of food consumed by the cut-off time was measured.

#### e. Isolation induced aggression

The isolation induced aggression test assesses territorial defense. After 20 days of housing in a single cage the test mouse had an encounter with a young intruder male under continuous video surveillance. The time to sniffing, boxing and biting were taken as indicators of investigation and aggression. If biting happened, the test was immediately terminated.

#### f. Morris swim navigation task

This task was used to test spatial learning and memory. The test was conducted in a white circular wading pool (diameter 120 cm) with a submerged platform (diameter 14 x 14 cm) 1.0 cm below the water of the pool in a fixed location to serve escape from the water. Temperature of the water was kept at 20 ± 0.5°C. The location of the hidden platform was kept constant and the starting position varied between four different locations at the pool edge, with all mice starting from the same position in a given trial. Each mouse was placed in the water with its nose pointing towards the wall. If the mouse failed to find the escape platform within 60 s, it was placed on the platform for 10 s by the experimenter (the same time was allowed to stay for mice that found the platform). The acquisition phase consisted of five daily trials with a 10 min interval across four days and three training trials on the 5th day. The first and the last trial of the 5th day were run without the platform to determine an eventual search bias as an index of spatial memory. The mouse was video-tracked and the video analysis program calculated the escape latency, swimming speed, path length and time in the pool periphery (10 cm from the wall) and the platform zone (diameter 30 cm).

#### g. Passive avoidance task

This task was used as a control for long-term memory, since in contrast to Morris swim task, increased activity level leads to impaired performance in this task. The mouse was placed in the well-lit side of a two-compartment box and was freely allowed to enter the dark, closed compartment through a hole in the dividing wall. As soon as the mouse entered the dark side, the slide door separating the compartment was closed and a mild foot-shock delivered (shock parameters: 0.30mA current intensity and two times for 2s). The mouse was then taken to its home cage. The memory for the aversive experience was assessed 48 h later by taking the time for the mouse to enter the dark compartment with a cut-off time of 180s.

#### Immunohistochemistry

Histological examination was done on 10 sham-exposed mice and 10 mice exposed to the 120 μT MF. The mice were deeply anesthetized with pentobarbiturate-chloralhydrate cocktail and transcardially perfused with ice-cold saline to rinse blood from the brain. One hemibrain was immersion fixed in 4% paraformaldehyde, for 4 h, dehydrated in 30% sucrose overnight and stored in a cryoprotectant at– 20 ^o^C until processed for histology (the other hemibrain was used for mRNA analysis). Coronal 35 μm slices were cut, and three sections of the septal hippocampus (spacing 200 μm) were stained for glial fibrillary acidic protein (GFAP) primary antibody (1:1000; Sigma) to visualize activated astrocytes.

Similarly, three sections each 200 μm apart starting from septal hippocampus were stained with doublecortin antibody (1:1,000; Santa Cruz, sc-8066) to visualize newly-born neurons. Corresponding biotinylated rabbit, anti-goat secondary antibodies (1:500) were used followed by incubation with HRP-labeled Streptavidin (1:1000; GE Healthcare). Color was developed by reacting nickel ammonium sulfate diaminobenzidine with hydrogen peroxide. Reaction was stopped with phosphate buffer and sections were then mounted, cleared in xylene, and coverslipped.

To determine GFAP immunoreactivity in the hippocampus, photomicrographic images of the hippocampal regions were taken using a camera (AxioCam ERc5sm) connected to a microscope (Zeiss Axio Imager, M2). The optic density of the entire hippocampal cross section was subtracted from a reference area in the somatosensory cortex in each section and averaged across the three sections.

#### RNA extraction and quantitative PCR (qPCR) analysis

Hippocampus were dissected from one hemibrain and snap frozen in liquid nitrogen until collection of tissues from all animal then stored at –70 ^o^C until the mRNA expression analysis. RNA was extracted from hippocampal tissue of sham exposed (n = 10) and 120 μT (n = 20) exposed mice using Direct-zol RNA MiniPrep (Zymo Research) RNA extraction kit according to manufacturer’s protocol. The synthesis of cDNA was carried out using SuperScript III First-Strand Synthesis System for RT PCR (LifeTechnologies). PCR primers specific for mouse *BDNF* (5’-TGGCTGACACTTTTGAGCAC-3’ and 5’-GTTTGCGGCATCCAGGTAAT-3’), mouse *TNFα* (5’-CGAGTGACAAGCCTGTAGCC-3’ and 5’-GTGGGTGAGGAGCACGTAGT-3’), and mouse *GAPDH* (5’AACTTTGGCATTGTGGAAGG-3’ and 5’-ACACATTGGGGGTAGGAACA-3’) were used for the qPCR. The qPCR reaction was performed using FastStart SYBR Green Master (Roche) Stratagene Mx3000P (Aligent Technologies). The comparative ΔΔCt method was used to calculate *GAPDH*-normalized expression levels of brain-derived neurotrophic factor (BDNF) and tumor necrosis factor alpha (TNFα).

## Statistical analysis

All statistical analyses were done using IBM-SPSS 21.0 for Windows. The results are presented as mean±SEM. All test results were normalized to the mean of the sham-exposed group of each cohort. Oneway-ANOVA was used in tests that included only one time point. Spontaneous explorative activity, passive avoidance task and the Morris swim task were analyzed with ANOVA for repeated measures, with the test day as the within-subject factor. Dunnett’s test was run as the post-test with the sham group as comparison in case of a significant main ANOVA result. Analysis of the histological and gene expression data was done by comparing two groups with the independent samples t-test. The threshold for significance was set at p < 0.05.

## Results

### Body weight

**Fig 1:**
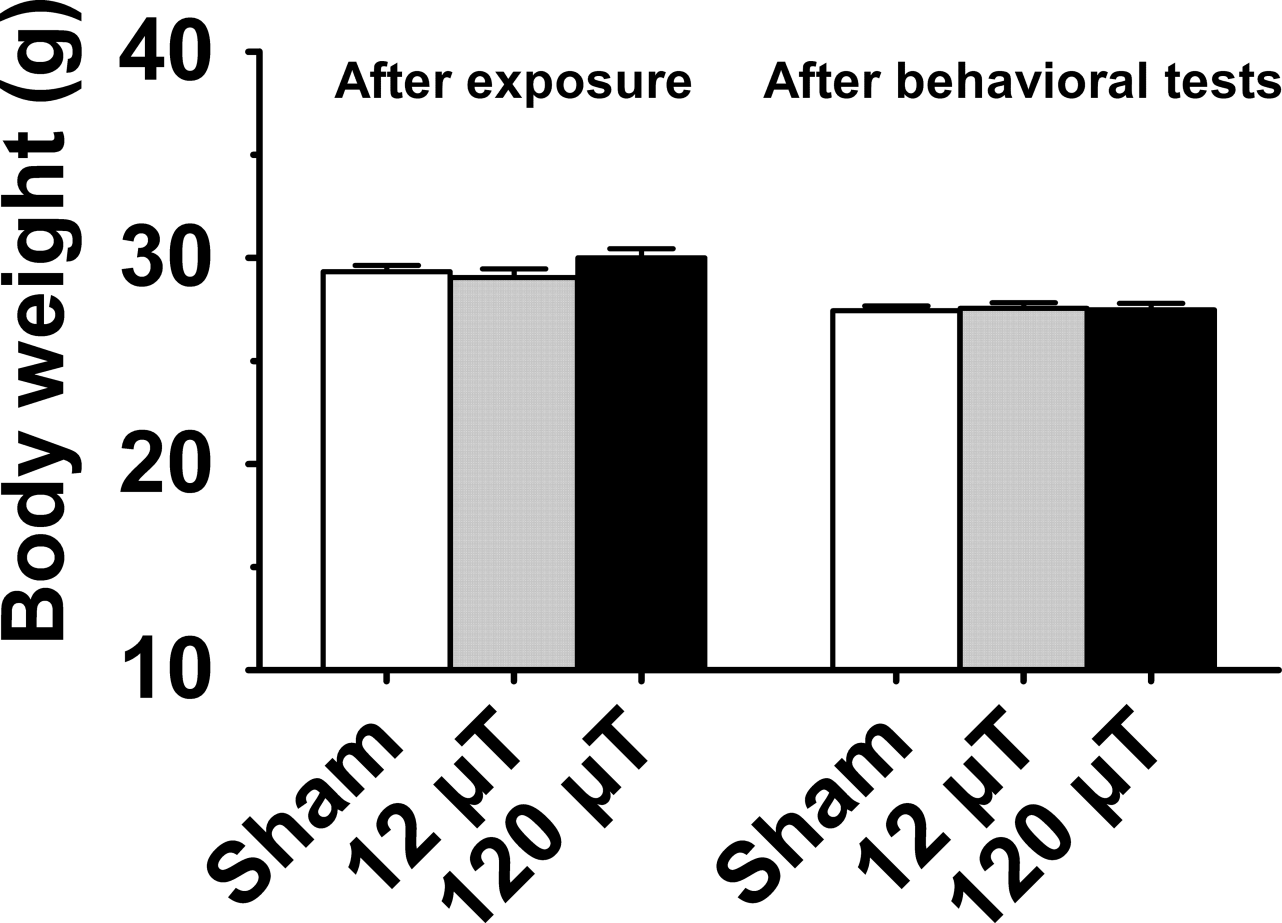
Body weight of male mice exposed to 7.5 kHz magnetic fields at 12 or 120 μT. Data are presented as group mean±SEM. (n = 30 in the sham exposed group, n = 20 in each exposed group).

As a measure of general health we determined the body weights of the animals after the completion of exposure (animals were ear-marked only after the exposure to avoid potential triggering for fighting in the group cage) and after behavioral tests. The body weights did not differ between the sham-exposed and IF MF exposed groups (p = 0.98; Fig 1). There were no changes in body weight after behavioral test (p = 0.97).

### Behavioral tests

Summary of the results of the behavioural test is presented in S1 Table. The results of individual tests are described below.

#### a. Spontaneous exploratory activity

**Fig 2:**
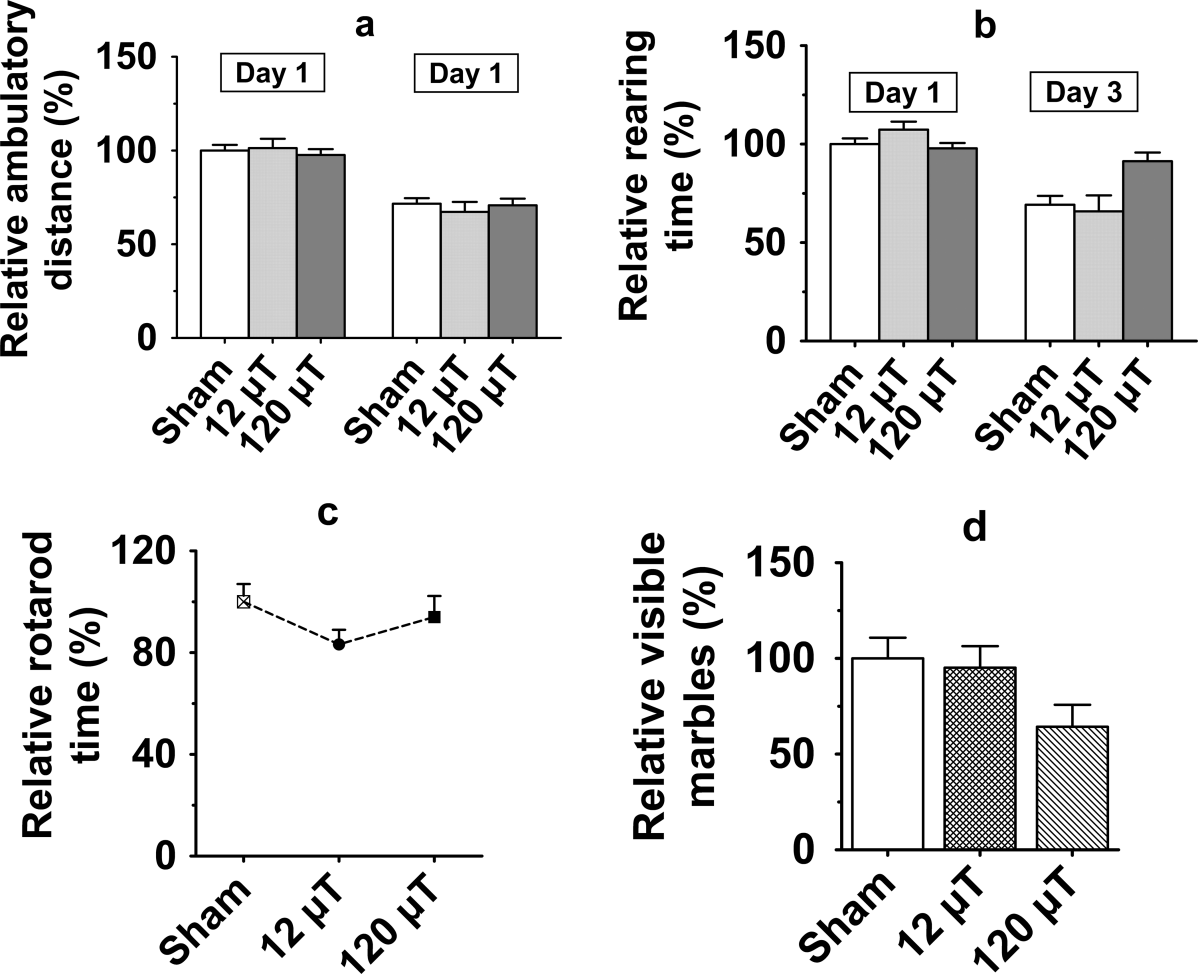
Motor performance in male mice after exposure to 7.5 kHz magnetic fields at 12 or 120 μT. (a) Spontaneous exploratory activity; Relative ambulatory distance (gross horizontal locomotion) (b) Relative time spent for rearing in a novel test cage during 10-min free exploration (c) Relative latency to fall off from the Rotarod. (d) Marble burying test for neophobia. The y-axis values represent unburied marbles. To control for between-cohort differences, relative values (mean±SEM) are shown in relation to the value observed in the sham-exposed group of each cohort. (n = 20 in each exposed group, n = 30 in the sham-exposed group, except in the Rotarod test, in which n = 20 in the sham-exposed and 120 μT groups, n = 10 in the 12 μT group).

Mice were first tested in an automated activity monitor to detect IF MF effects on spontaneous exploratory activity. The spontaneous activity did not differ between the exposure groups in terms of ambulatory distance (gross horizontal locomotion; p = 0.92; Fig 2A), time engaged in stereotypic movements (p = 0.47) or time spent in rearing (p = 0.18; Fig 2B). There was no session by exposure interaction.

#### b. Motor coordination and balance

To further assess possible effects on motor coordination and balance, we tested the ability of mice to stay on an accelerating rotating rod (Rotarod). The latency to fall off did not differ between the exposure groups (p = 0.40; Fig 2C).

#### c. Marble burying task for neophobia

Mice at the highest exposure group tended to leave fewer marbles visible than other mice but the overall difference between the groups was not significant (F _2, 67_ = 2.77; p = 0.07; Fig 2D).

#### d. Novelty suppressed feeding test

The exposure groups did not differ either in the latency to sniff (p = 0.69) or to bite (p = 0.97) the food pellet.

#### e. Isolation-induced aggression

There was no difference in the number of aggressive contacts between the sham and IF MF exposed groups (p = 0.80).

#### f. Morris swim task

**Fig 3:**
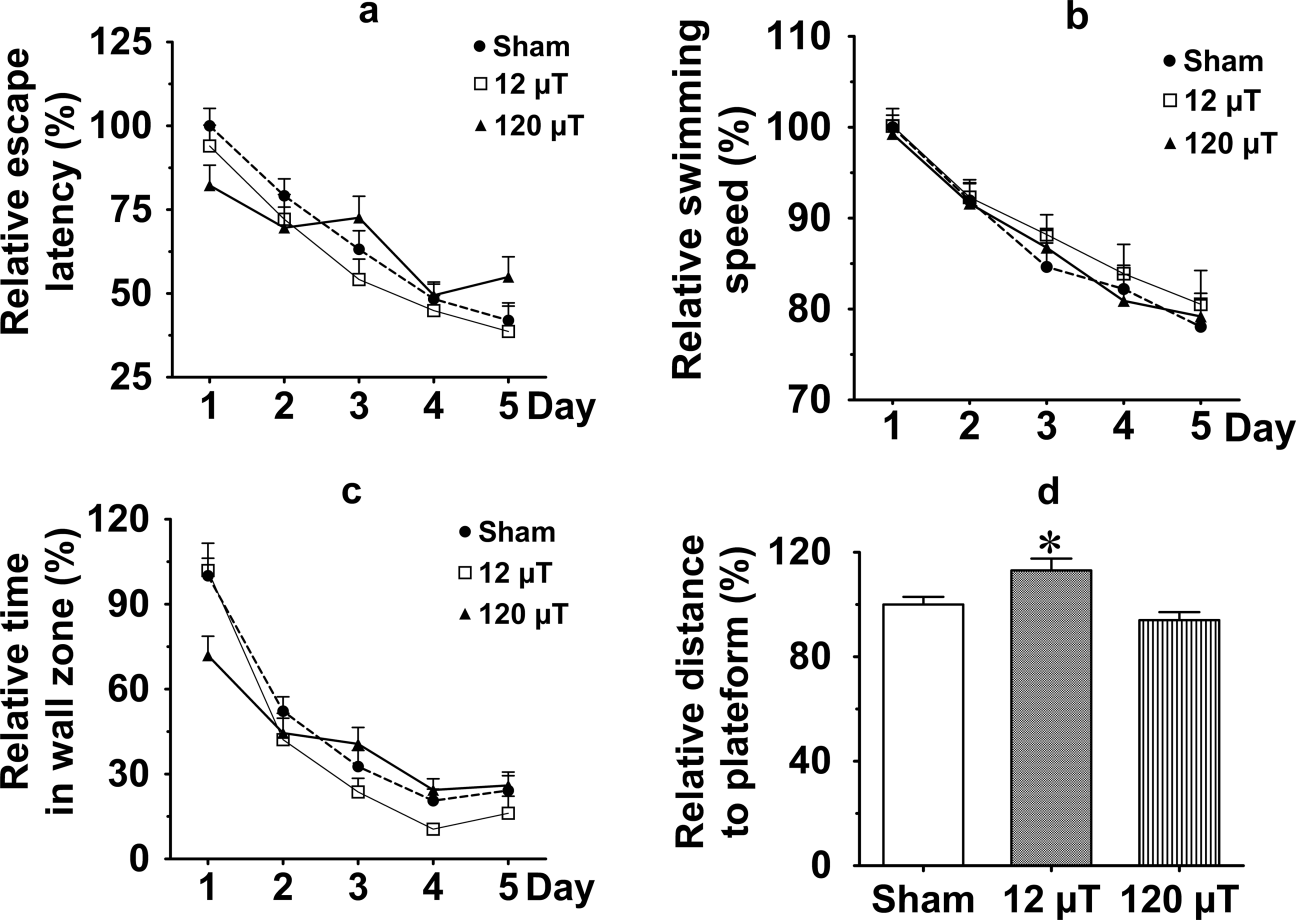
Assessment of spatial learning and memory in the Morris swim task in male mice after exposure to 7.5 kHz magnetic fields at 12 or 120 μT. (a) Relative escape latencies during 5 days of task acquisition. (b) Relative swimming speed. (c) Relative time spent in the pool wall zone. (d) Relative search bias during the probe trial on day 5 as a mean distance to the platform. To control for between-cohort differences, relative values (mean±SEM) are shown in relation to the value observed in the sham-exposed group of each cohort (n = 30 in sham exposed group, n = 20 in each exposed group). * Significant difference from the sham group, p < 0.05 (Dunnett’s test).

The Morris swim task was used to test spatial learning and memory. No main effect on escape latency was observed in ANOVA for repeated measures (ANOVA-RM, p = 0.68). However, the day by exposure interaction was significant (F _8, 268_ = 2.3; p = 0.019). This resulted from a smaller decrement in escape latency from day 1 to day 5 in the 120 μT group than in the other groups (Fig 3A), suggesting impaired spatial learning. Notably, swimming speed (Fig 3B) was not affected by IF MF (p = 0.80).

Typically, mice first try to find an escape through the pool wall, and gradually give up this fruitless strategy over the days. To obtain a measure of learning a better strategy in the task, we also measured the time spent in the pool periphery (10 cm from the wall). Like in the case of escape latency, no main effect on time spent in periphery was observed in ANOVA-RM (p = 0.41), but the day by exposure interaction was significant (F _8, 268_ = 3.6; p = 0.001), as the decrement from day 1 to day 5 was smaller in the 120 μT group than in the other groups (Fig 3C). This finding also speaks for impaired learning in the high-exposure group.

Finally, we assessed the search bias during a probe trial without the platform on the last trial on day 5 (Fig 3D). There was a significant difference between the groups (F _2, 69_ = 6.80; p = 0.002), such that the 12 μT group kept a longer distance to the platform than the sham group (p = 0.017), while the 120 μT group did not differ from the sham group (p = 0.38). The search bias is considered the best measure of memory of the spatial location, while escape latency and time in the wall zone are considered more general indices of learning. Therefore, having slightly different results on these parameters is not unusual.

#### g. Passive avoidance

**Fig 4:**
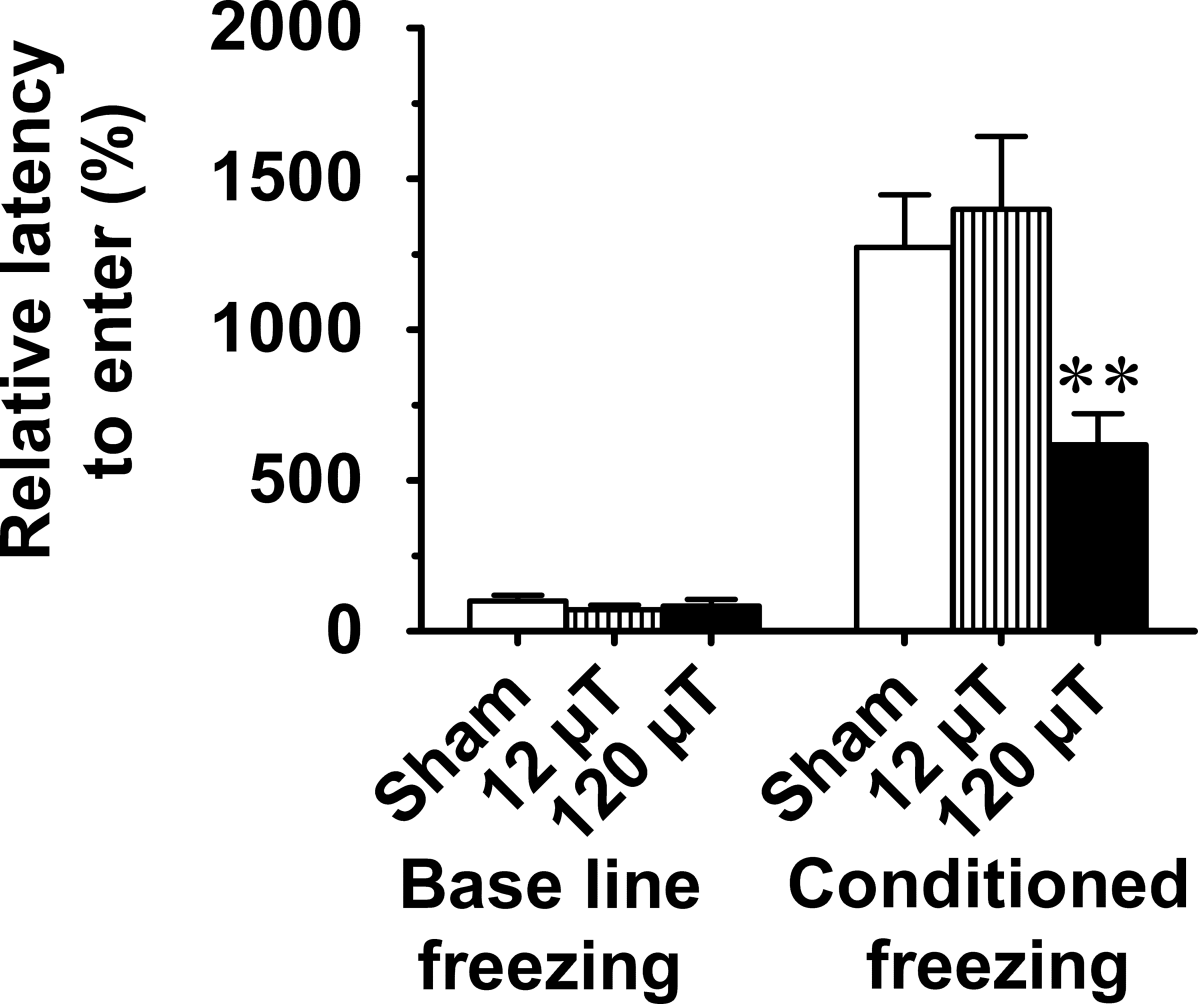
Passive avoidance task for long-term memory in male mice after exposure to 7.5 kHz magnetic fields at 12 or 120 μT. Relative latency (s) to enter the dark compartment 48 h after the learning session interval is shown. To control for between-cohort differences, relative values (mean±SEM) are shown in relation to the value observed in the sham-exposed group of each cohort (n = 30 in sham exposed group, n = 20 in each exposed group). ** Significant difference from the sham group, p < 0.01 (Dunnett’s test).

This task is among the oldest learning and memory tasks used in rodents and sensitive to lesions of the amygdala and hippocampus among other brain structures [16]. The latency to enter the dark compartment after a retention interval of 48 h was significantly shorter in the 120 μT group than in sham-exposed mice (F _2, 67_ = 4.6; p = 0.013; Fig 4) suggesting exposure-related impairment of memory over days.

#### Immunohistology

**Fig 5:**
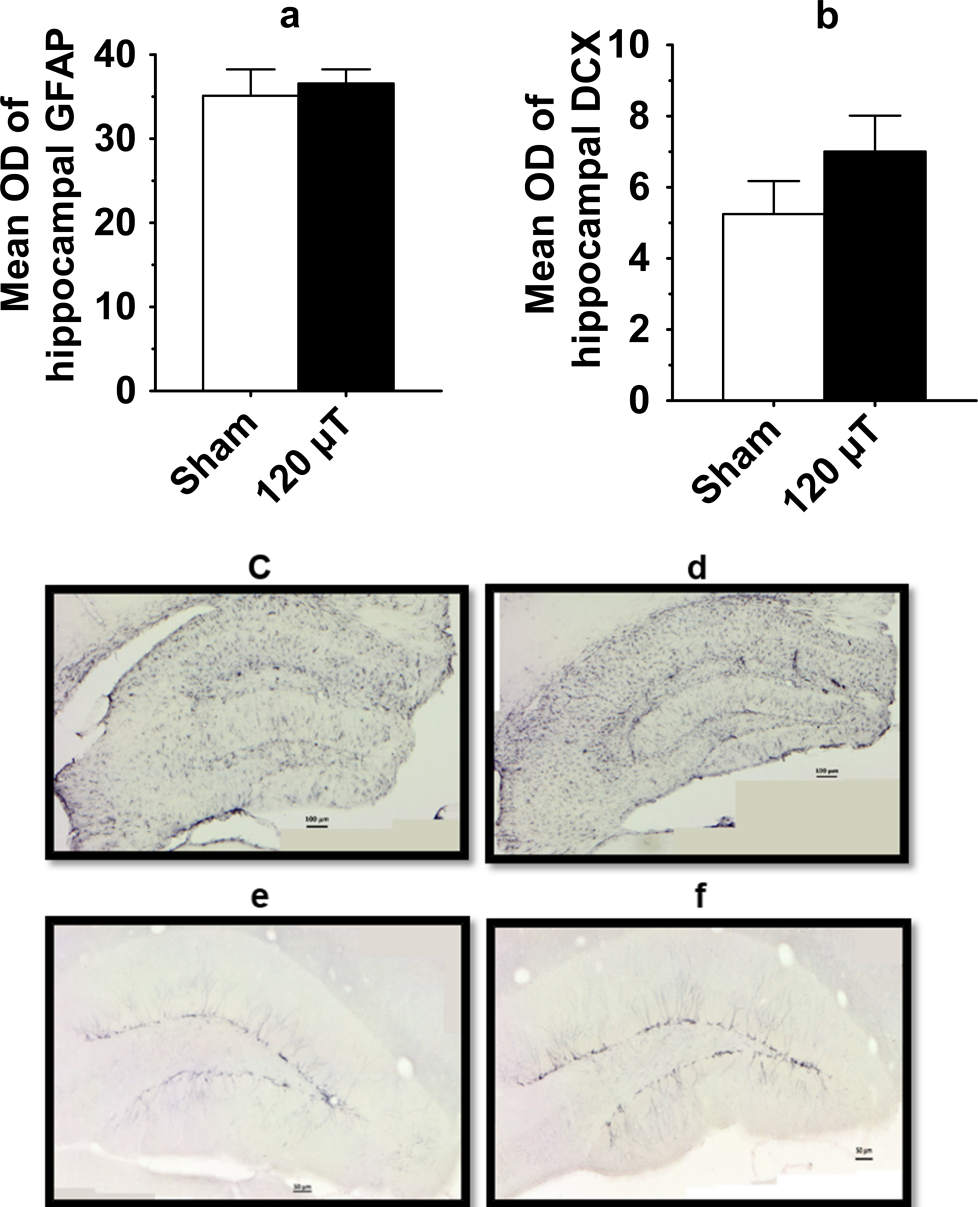
Histological analysis of the coronal sections of the septal hippocampus in male mice after exposure to 7.5 kHz magnetic fields at 120 μT. (a) The mean optic density (OD) of hippocampus stained for glial fibrillary acidic protein (GFAP) to visualize activated astrocytes. (b) The mean optic density of hippocampal doublecortin (DCX) staining to visualize newly-born neurons. The GFAP and DCX data are presented as mean±SEM (n = 10 in each group). (c, d) Representative photomicrographs of astrocytic GFAP immunostaining in hippocampus. Scale bar = 100 μm. (e, f) Representative photomicrographs of hippocampal DCX staining. Scale bar = 50 μm.

To assess eventual astrocyte activation in response to the IF MF exposure, we stained hippocampal sections for GFAP. The mean optical density of hippocampal GFAP staining did not differ between the exposure groups (p = 0.68, Fig 5A, 5C and 5D). Further, to assess whether long-term IF MF exposure affects hippocampal neurogenesis, we quantified doublecortin (DCX) immunoreactivity in the dentate gyrus of the hippocampus. Due to the high density of DCX-positive neurons and their neurites, we could not count the number of positive cells but analyzed the optic density of immunopositivity of the entire hippocampal cross section. The mean optic density of hippocampal DCX staining did not differ between the exposure groups (p = 0.22; Fig 5B, 5E and 5F).

#### qPCR analysis

**Fig 6:**
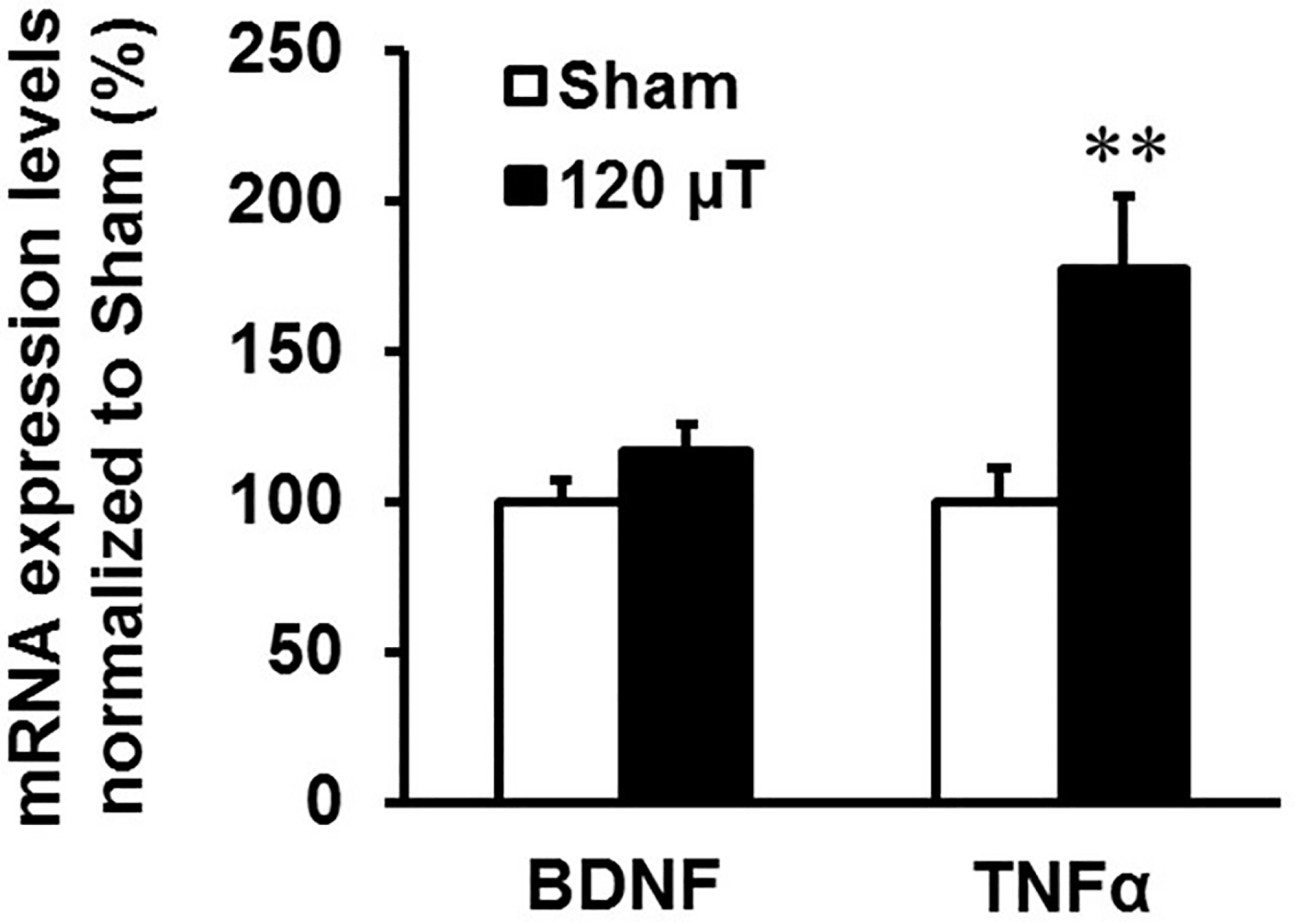
Expression analysis of brain-derived neurotrophic factor (BDNF) and tumor necrosis factor alpha (TNFα) in the hippocampus in male mice after exposure to 7.5 kHz magnetic fields at 120 μT. GAPDH normalized levels (mean±SEM) are shown (n = 10 in the sham exposed group, n = 20 or 18 (TNFα) in the 120 μT group). ** Significant difference from the sham group, p < 0.01 (t-test).

To elucidate the expression status of neuronal- and microglia-linked targets, the RNA levels of BDNF and TNFα were analyzed in the hippocampus of the sham-exposed and 120 μT groups using qPCR. Mice exposed at 120 μT showed a statistically significant increase in the GAPDH normalized levels of TNFα (p = 0.007), but not BDNF (p = 0.10) in comparison to the sham-exposed mice (Fig 6).

## Discussion

To the best of our knowledge, the present study is the first one to evaluate behavioral effects of long-term IF MF exposure in mice. We observed no signs of impaired general health in the exposed mice, and the body weights did not differ between the exposure groups. A large battery of behavioral tests commonly used in neuropharmacology, encompassing tests for motor activity, motor coordination, anxiety and aggression revealed no deleterious effects of the exposure either. However, performance of the animals in the Morris swim task and in the passive avoidance task indicated moderate impairment of spatial learning and memory in the high-exposure group. Immunohistochemical analysis did not reveal significant effects on astroglial activation or neurogenesis in the hippocampus. However, mRNA expression of the main proinflammatory cytokine TNFα was elevated in the high-exposure group. Collectively, these findings suggest that exposure to a 7.5 kHz, 120 μT MF for 5 weeks may be associated with some undesired effects on spatial learning and memory consolidation, which are among of the most sensitive measures of brain function.

The Morris swim task (‘water maze’) is considered a standard test for hippocampal dependent spatial learning and memory [14]. However, it is a complex task, and impairment especially in the acquisition phase may stem from many reasons [17]. Importantly, the IF MF exposure did not affect the swimming speed, which is the most common confound in the test. We assessed the learning phase by two independent parameters. The most common measure of learning, escape latency, improved less with time in the 120 μT group than in the other groups (significant time by exposure interaction), suggesting that learning was impaired in this group. To assess typical wall-clinging behavior of mice in the task, we also measured the time spent in the pool periphery. There was again a significant time by exposure interaction, such that the 120 μT group showed less improvement with time than the control group. This suggests that mice of the higher exposure groups did not adopt an efficient search strategy at the same rate as the control group. Impairment in the task acquisition was limited to the 120 μT group. The last test day included a probe test without the platform to directly assess the search bias of the mice. In contrast to rats, the mice do not show a clear focused search pattern in the near vicinity of the former platform location. Therefore, the average distance to the platform has been considered the best parameter to describe the search bias in mice [18]. There was a significant difference between the groups, such that the 12 μT group kept a longer distance to the platform than the sham group, while the 120 μT group did not differ from the sham group. This finding does not support a dose-dependent impairment in spatial memory. The passive avoidance task indicated a clear impairment of memory over days in the 120 μT group. This task is also sensitive to hippocampal lesions [16] but in contrast to the Morris swim task, it measures a single-trial learning. Importantly, changes in the activity level have opposite biases in these two tasks, further emphasizing the fact that the impaired performance of the 120 μT group likely reflects memory impairment rather than changes in motor performance.

There are no previous studies on behavioral effects of IF MFs, but two studies have measured brain biomarkers and morphology of the hippocampus in IF MF-exposed mice [19, 20]. Mice were exposed to 21 kHz MFs at 3.8 mT, 1 h/day for 2 weeks and the hippocampal mRNA expression level were evaluated for several memory related genes: NMDA receptor subunits NR1, NR2A and NR2B; key signal transduction pathway genes CaMKIV and CREB; and neurotrophins NGF and BDNF. There were no significant alterations in expression levels of these genes in the IF MF exposed group as compared to the control group. Further, a morphological analysis of hippocampal dentate gurys using hematoxylin and eosin staining did not reveal exposure-related changes [19]. More recently, the same group studied similar memory related genes and also the immediate early gene cFos and immunological markers IL 1β, TNF-α, and COX2, and the oxidative stress marker HO1 in the hippocampus of 3- and 7-week-old male mice. The mice were exposed *in utero* and during postnatal development to 21 kHz, 3.8 mT MFs for 1 h/day [20]. Changes in the MF-exposed group were observed in hippocampal gene expression of cFos, NMDA subunits NR1 and, NR2B, signaling pathway molecules CaMKIV, and CREB1, inflammation markers IL 1β, COX2, and TNF-α, and the oxidative stress marker HO1 in 7-week-old mice but not in 3-week-old mice. Histological examination of hippocampal DG stained by hematoxylin and eosin did not reveal any morphological changes, and immunohistochemical analysis of microglia for Iba-1 did not show activation. Importantly, no effects were observed in animals that were allowed to recover for one day after termination of exposure, indicating that these effects were transient. Our results cannot be directly compared to these previous studies, since the frequency and magnetic field strength were different, and the animals were exposed only for 1 h/day in contrast to the nearly continuous exposure used in the present study.

Although previous data on behavioral and central nervous system (CNS) effects are scarce for IF MFs, several previous studies have addressed such effects in animals exposed to ELF MFs at 50 or 60 Hz. As there is little understanding of the frequency dependency of nervous system effects of low intensities MFs, studies on ELF field effects are briefly discussed here in order to identify possible similarities with the findings of the present study. A few studies have reported effects from short-term exposure to ELF MFs. Exposure to 50 or 60 Hz MFs at 0.75 to 1 mT for 45 min immediately before each training session impaired spatial learning in rats and mice [21–23]. The threshold for these effects appeared to be between 7.5 and 75 μT [24]. A single 4-h exposure to a 50 Hz MF at 8 mT after the learning session impaired the memory consolidation in mice tested by passive avoidance task [25]. In human volunteers exposed to 45 Hz MFs at 1.26 mT for 15 min or 1 h, practice effects on reaction time (decrease of reaction time in repeated test sessions) was suppressed by the MF exposure [26, 27]. Several other studies have used continuous or repeated daily exposures to ELF MFs for longer periods. Exposure of mice to 50 Hz MFs at 1 mT for 4h/d for 12 weeks was reported to lead to impairment of spatial learning and memory and increased hippocampal and striatum oxidative stress, while no effects were observed at 0.1 mT [28]. Another study reported impaired recognition memory as tested by the novel object recognition test in mice exposed to 50 Hz MFs at 1 mT for 7–10 days, 12 h/d [29]. No effects were reported at 0.4 or 0.6 mT or if the exposure duration was less than 7 days or more than 10 days. On the other hand, improved spatial learning and memory has been reported in other studies in rats and mice exposed to 50 Hz MFs at 1–2 mT for up to 4 weeks, 1–4 h/d [30–32].

Studies using 50 Hz MFs between 100 and 500 μT (from 14 days, 4h/d to 24 weeks, 20 h/d) have not found any effect on spatial learning and memory or brain morphology in rats [33–35].

Overall, there is some evidence that the central nervous system is affected by ELF MFs, particularly if the field strength is 1 mT or higher. However, the findings are variable (possibly depending on varying experimental details), and their generalizability to higher frequency MFs, such as the 7.5 kHz fields used in the present study, are not known at present.

## Conclusions

Impaired task acquisition in the Morris swim task and decreased latency in the passive avoidance task suggest negative effects of 7.5 kHz exposure at the 120 μT on learning and memory. Also, expression of proinflammatory cytokine TNFα was significantly increased in the 120 μT group. These findings suggest that exposure to a 7.5 kHz, 120 μT MF may lead to mild learning and memory impairment possibly through an inflammatory reaction in the hippocampus. In contrast, the exposure did not affect general health, body weight, spontaneous activity, motor coordination, anxiety or aggression, and no exposure-related histopathological changes or expression of BDNF were observed. Further research is warranted to increase understanding of the reproducibility, mechanisms and human health implications of the results.

## Supporting information

S1 Table Summary of the behavioral results expressed as absolute values. (DOCX)
