# Supplementary material for: Behavioral testing of mice exposed to intermediate frequency magnetic fields indicates mild memory impairment
Source: PLoS One. 2017 Dec 4;12(12):e0188880. doi: 10.1371/journal.pone.0188880 (PMC5714647; doi:10.1371/journal.pone.0188880)
Supplement: S1 Table — (DOCX) [file pone.0188880.s001.docx]

**Behavioral testing of mice exposed to intermediate frequency magnetic fields indicates mild memory impairment**

Kajal Kumari^1*^, Hennariikka Koivisto^2^, Matti Viluksela^1,3^, Kaisa M. A. Paldanius^4^, Mikael Marttinen^4^, Mikko Hiltunen^4^, Jonne Naarala^1^, Heikki Tanila^2^, Jukka Juutilainen^1^

**Supplementary Table S1. Summary of the behavioral results expressed as absolute values.**

|  | Cohort 1 |  | Cohort 2 |  | Cohort 3 |  |
| --- | --- | --- | --- | --- | --- | --- |
| Test parameter | Sham (n =10) | 12 μT (n = 10) | Sham (n = 10) | 12 μT (n = 10) | Sham (n = 10) | 120 μT (n = 20) |
|  | **Mean±SEM** | **Mean±SEM** | **Mean±SEM** | **Mean±SEM** | **Mean±SEM** | **Mean±SEM** |
| 1. Spontaneous exploratory activity |  |  |  |  |  |  |
| Ambulatory distance (cm) | 1771±84 | 1429±63 | 1738±78 | 2030±128 | 1977±125 | 1924±65 |
| Stereotypic movements(s) | 35±3 | 49±5 | 36±3 | 27±3 | 25±4 | 39±2 |
| Rearing time(s) | 86±4 | 74±2 | 84±6 | 111±5 | 267±11 | 261±9 |
| 2. Rotarod |  |  |  |  |  |  |
| Latency to fall (s) | NA | NA | 215±17 | 179±12 | 157±18 | 148±13 |
| 3. Marble burying (visible marbles)(amount) | 4.2±0.7 | 3.70±0.7 | 4.6±0.9 | 4.7±0.8 | 3.5±0.8 | 2.2±0.4 |
| 4. Novelty suppressed feeding test |  |  |  |  |  |  |
| Latency to bite (s) | 84±25 | 108±26 | 67±18 | 45±06 | 133±32 | 126±18 |
| 5. Isolation-Induced Aggression |  |  |  |  |  |  |
| Latency to sniff (s) | 19±04 | 21±06 | 12±02 | 15±03 | 48+±6 | 49±4 |
| 6. Morris swim task |  |  |  |  |  |  |
| Escape latency, day 1 (s) | 37±3 | 35±4 | 33±4 | 35±4 | 38±3 | 31±2 |
| Escape latency, day 5 (s) | 13±3 | 18±5 | 9±2 | 10±2 | 24±3 | 21±2 |
| Swimming speed, day 1 (cm/s) | 23±0.6 | 23±0.4 | 22±0.6 | 22±0.7 | 23±0.4 | 23±0.4 |
| Swimming speed, day 5(cm/s) | 20±0.9 | 16±1 | 18±0.8 | 19± 0.7 | 14±0.9 | 18±0.6 |
| Probe trial (Search bias) |  |  |  |  |  |  |
| Distance to the platform (cm) | 36±2 | 39±3 | 34±1 | 40±2 | 42±3 | 40±1 |
| 7. Passive avoidance |  |  |  |  |  |  |
| Latency to enter(s) | 76±20 | 70±20 | 106±21 | 117±23 | 65±17 | 36±06 |
| NA = test was not performed in Cohort 1 | | |  |  |  |  |
